# Exercise for rotator cuff tendinopathy

**DOI:** 10.47626/1679-4435-2022-698

**Published:** 2023-02-03

**Authors:** Jeffeson Hildo Medeiros de-Queiroz, Marisa Barreto de-Medeiros, Rosângela Nascimento de-Lima, Denilson de Queiroz Cerdeira

**Affiliations:** 1 Departamento de Fisioterapia, Universidade Federal do Ceará, Fortaleza, CE, Brazil; 2 Departamento de Enfermagem, Faculdade do Vale do Jaguaribe, Aracati, CE, Brazil; 3 Departamento de Enfermagem, Faculdade Maurício de Nassau, Fortaleza, CE, Brazil; 4 Departamento de Fisioterapia, Faculdade do Vale do Jaguaribe, Aracati, CE, Brazil

**Keywords:** tendinopathy, rotator cuff injuries, exercise therapy, shoulder pain, International Classification of Functioning, Disability and Health., tendinopatia, lesões do manguito rotador, terapia por exercício, dor de ombro, Classificação Internacional de Funcionalidade, Incapacidade e Saúde.

## Abstract

Rotator cuff tendinopathy is among the main causes of shoulder pain. It is characterized by lesions without rupture caused by overload, work-related repetitive strain injury, or metabolic changes such as diabetes affecting one or more tendons, which cause pain, morphological alterations, and disability. This study aimed to evaluate the effects of exercise-based therapy on shoulder pain reduction and functioning improvement in patients with rotator cuff tendinopathy. This was a systematic review. Data were collected from randomized controlled trials retrieved from PubMed, Biblioteca Virtual em Saúde, PEDro, Web of Science, Scopus, and CENTRAL metasearch engines. The PEDro scale was used to evaluate the methodological quality of the selected studies. Eccentric exercise, conventional exercise, scapular and rotator cuff muscle strengthening, rotator cuff strengthening plus pectoralis major strengthening, high-load training, and low-load training were effective for the outcomes investigated in this study. Furthermore, goniometry, visual analogue scales, the Constant Murley score, the Disabilities of the Arm, Shoulder and Hand questionnaire, and the Shoulder Pain and Disability Index were constantly used to measure pain and functioning. Therapeutic exercises should be performed in this population, and new randomized controlled trials should be conducted with the goal of achieving the same outcome. The International Classification of Functioning, Disability and Health should be increasingly used in studies addressing patient functioning.

## Abstract (pt)

Entre as causas de dor no ombro, as tendinopatias do manguito rotador são as mais comuns. São conceituadas como lesões sem ruptura causadas por sobrecarga, esforço repetitivo em atividades laborais ou alterações metabólicas, como o diabetes, que afetam um ou mais tendões, causando dor, alteração morfológica e incapacidade. Objetiva-se avaliar os efeitos do tratamento baseado em exercícios para a redução da dor no ombro e melhora da funcionalidade de pacientes com tendinopatia do manguito rotador. Trata-se de uma revisão sistemática. Os dados foram coletados a partir de ensaios controlados aleatorizados encontrados nos metabuscadores PubMed, Biblioteca Virtual em Saúde, PEDro, Web of Science, Scopus e CENTRAL. A escala PEDro foi utilizada para avaliar a qualidade metodológica dos estudos. Exercício excêntrico, exercício convencional, fortalecimento dos músculos escapulares e do manguito rotador, fortalecimento do manguito rotador com adição de fortalecimento de peitoral maior, treino de alta intensidade e treino de baixa intensidade foram eficazes para os desfechos investigados neste estudo. Ademais, percebeu-se que a goniometria, a escala visual analógica e os questionários Constant Murley, Disabilities of the Arm, Shoulder and Hand e Índice de Dor e Incapacidade no Ombro são ferramentas constantemente utilizadas para mensurar a dor e funcionalidade desses pacientes. Sugere-se que o exercício terapêutico seja realizado nessa população e que novos ensaios controlados aleatorizados sejam realizados visando a esse mesmo desfecho. Além disso, sugere-se uma maior utilização da Classificação Internacional de Funcionalidade, Incapacidade e Saúde nos estudos que abordam a funcionalidade desses pacientes.

## INTRODUCTION

The shoulder joint is a functional complex formed by bones, muscles, cartilage, tendons, capsules, bursae, and ligaments that unite the upper limbs to the trunk, forming the shoulder girdle. One of its functions is to allow the upper limb to move with great amplitude (180°), ensuring a considerable diversity of movements, such as flexion, extension, internal rotation, and external rotation.^^1^,^2^^

Among the anatomical structures that compose the shoulder complex, the tendons have a morphology rich in connective tissue that attaches muscle to bone, enabling stability, movement, and force transmission from muscle to bone. Tendinopathies are characterized by injuries without rupture caused by overload, work-related repetitive strain injury, or metabolic alterations such as diabetes affecting one or more tendons, which cause pain, morphological alterations, and inability to perform sports and/or activities of daily living.^^2^-^4^^ Rotator cuff tendinopathy is among the main causes of shoulder pain.^^3^,^5^^

With the goal of achieving pain reduction and increased functioning in patients with shoulder tendinopathy, conservative treatment with therapeutic exercise may be an alternative way of caring for these patients. Although several randomized controlled trials (RCTs) have been conducted over the years to further understand this issue, systematic reviews of exercise-based interventions and common outcomes related to rotator cuff tendinopathy are currently lacking.^^6^^ Therefore, the objective of this study was to evaluate the effects of exercise-based treatment on shoulder pain reduction and functioning improvement in patients with rotator cuff tendinopathy.

## METHODS

### TYPE OF STUDY

This integrative review of RCTs followed the Preferred Reporting Items for Systematic Reviews and Meta-Analyses (PRISMA) statement,^^7^^ which is destined for systematic reviews with and without meta-analysis. Systematic reviews are secondary studies aimed at compiling and critically evaluating similar studies on a topic.^^8^^ This systematic review evaluated the effects of therapeutic exercise on pain reduction and functioning improvement in patients with rotator cuff tendinopathy.

### SEARCH STRATEGY

The research question was elaborated according to the PICO strategy (patient [P], intervention [I], comparison [C], and outcome [O]). Articles were searched on PubMed, Scopus, Web of Science, and Biblioteca Virtual em Saúde metasearch engines with no restrictions on date of publication. The Phisiotherapy Evidence Database (PEDro) and Cochrane Central Register of Controlled Trials were also searched for RCTs. The CAFe system was used to access restricted databases on the Coordenação de Aperfeiçoamento de Pessoal de Nível Superior (CAPES) Journals website. Database search occurred from November 2019 to November 2020. We did not have to contact any of the authors of the retrieved/included studies because they were all available in full.

To search the databases and metasearch engines, the following Health Sciences Descriptors (DeCS) were used: (Tendinopathy OR Tendons) AND “Rotator Cuff” AND “Exercise Therapy” AND “Shoulder Pain”. The search was conducted according to the peculiarities of each database and metasearch engine. Additionally, the search strategy and study eligibility processes were conducted by two independent reviewers.

### ELIGIBILITY CRITERIA

Eligibility criteria were RCTs that answered our research question and included patients with shoulder dysfunction and pain due to rotator cuff tendinopathy, published in peer-reviewed scientific journals indexed in selected databases.^^9^^ RCTs that were not registered at ClinicalTrials.gov or the Brazilian Registry of Clinical Trials (REBEC) and that did not include at least one exercise-based intervention group or the outcomes investigated in this study were excluded. A total of 4 RCTs were included in this study. Duplicate publications were excluded with the help of a reference manager (Mendeley Desktop, version 1. 19.4).

### RISK OF BIAS ASSESSMENT

The PEDro scale was used to evaluate the methodological quality of the included studies.^^10^^ All RCTs were indexed in this scale through the PEDro database. The PEDro scale aims to assess the methodological quality of RCTs using 11 items scored from 0 to 10.^^11^^ The score was not used as an inclusion/exclusion criterion, but rather as an indicator of the methodological quality of the included RCTs.

### DATA ANALYSIS

Data were analyzed by two independent researchers. Meta-analysis was not conducted due to the heterogeneity of the studies.

## RESULTS AND DISCUSSION

The search strategy described in Table 1 retrieved 88 studies from the investigated databases and metasearch engines.

**Table 1:** Description of the studies retrieved from the databases

Database/metasearch engine	Retrieved studies	Included studies
PubMed	18	3
BVS	0	0
PEDro	0	0
Web of Science	68	1
Scopus	2	0
CENTER	0	0

BVS = Biblioteca Virtual em Saúde; PEDro = Phisiotherapy Evidence Database.

**Figure 1:**
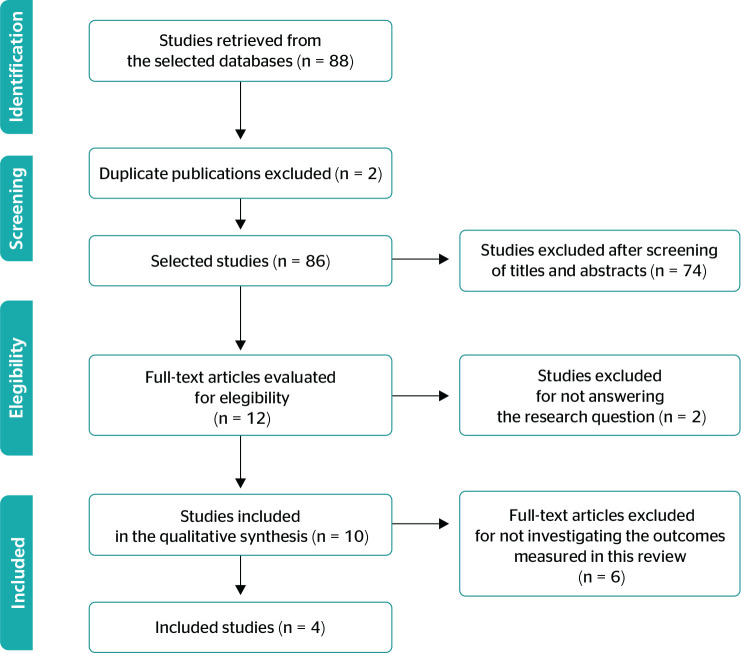
Application of the 2009 Preferred Reporting Items for Systematic Reviews and Meta-analysis (PRISMA) statement.

According to the flow diagram of study selection (Figure 1), 88 studies were retrieved from the databases using the selected keywords. Of the total, 2 duplicate articles were removed, resulting in 86 articles for title and abstract analysis. Seventy-four studies were excluded after the analysis. Of 12 included studies, 8 were excluded because they did not answer the research question and were not registered at ClinicalTrials.gov or REBEC.

The following techniques for pain reduction and functioning improvement in patients with rotator cuff tendinopathy were observed in the 4 RCTs included in this review: eccentric exercise, conventional exercise, scapular and rotator cuff muscle strengthening, rotator cuff strengthening plus pectoralis major strengthening, high-load training, and low-load training. Importantly, the RCTs analyzed in this study had a PEDro score of 7 and 8. Publication dates were from 2017 to 2019. Mean patient age ranged from 45.7 to 50.2 years. Symptoms lasted from 12.0 to 44.2 months (Table 2).

**Table 2:** Clinical and sociodemographic profiles of participants

Authors	Number of groups	Participant age	Duration of symptoms (months)
Dejaco et al.^^12^^	2 groups (isolated eccentric exercise vs conventional exercise).	Eccentric exercise (EE) group: 50.2 ± 10.8. Conventional exercise (CE) group: 48.6 ± 12.3	EE group: 16.9 ± 16.8. EC group: 23.1 ± 23.8
Boudreau et al.^^13^^	2 groups (scapular and rotator cuff muscle strengthening vs scapular and rotator cuff muscle strengthening plus coactivation with pectoralis major strengthening and latissimus dorsi)	Scapular and rotator cuff muscle strengthening (SRMS) group: 49.6 ± 13.2. Rotator cuff muscle strengthening plus coactivation with pectoralis major strengthening and latissimus dorsi (SRMS + PMLD) 50.2 ± 10.9.	SRMS group: 41.8 ± 40.5. RSMS + PMLD group: 44.2 ± 52.9.
Ingwersen et al.^^14^^	2 groups (high-intensity exercise vs low-intensity exercise)	High-intensity exercise (HIE) group: 45.7 ± 10.6. Low-intensity exercise (LIE) group: 46.5 ± 10.1	HIE group: 12.0. LIE group: 12.0.
Heron et al.^^15^^	3 groups (open chain resisted band exercises vs closed chain exercises vs minimally loaded range of movement exercises)	Open chain resisted band exercises (OC) group: 49.5. Closed chain exercises (CC) group: 50.4. Minimally loaded range of movement exercises (ROM) group: 49.8	OC group: 23. CC Blue group: 29. ROM Blue group: 26.

According to the data, study participants were aged > 45 years. This is in accordance with other studies in which patients diagnosed with tendinopathy started presenting symptoms after 40 years of age.^^5^^ Additionally, symptoms lasted an average of 12.0 to 44.2 weeks. The main study outcomes and results are described in Table 3.

**Table 3:** Description of studies analyzed in this systematic review

Authors	PEDro score	Intervention	Main study outcomes	Main results
Dejaco et al.^^12^^	7/10	36 patients with rotator cuff tendinopathy were randomly assigned to isolated eccentric exercise (n=20) vs conventional exercise (n=16). Both groups underwent a 12-week daily home-based exercise programme and received a total of 9 treatment sessions. A VAS was used to evaluate pain in participants. The Constant Murley questionnaire was used to evaluate range of motion and muscle strength.	Shoulder pain, shoulder range of motion, and isometric abduction strength in 45° in the scapular plane.	After the treatment period, there was a significant increase in the Constant Murley score and a significant decrease in VAS scores. No difference was found between the groups (p = 0.015) for any of the evaluated outcomes.
Boudreau et al.^^13^^	8/10	42 participants with rotator cuff tendinopathy were randomly assigned to scapular and rotator cuff muscle strengthening vs rotator cuff strengthening plus coactivation with pectoralis major and latissimus dorsi recruitment. The daily programs were performed at home for 6 weeks, with supervised training and 16 follow-up sessions. Functional limitations/symptoms (DASH - primary outcome - and the WORC index) and pain (VAS) were measured at baseline, 3 weeks, and 6 weeks.	Functional limitations/symptoms.	No significant group-by-time interaction was observed for the DASH questionnaire, WORC index, and VAS (p ≥ 0.55) Significant time effects were obtained for the WORC index and VAS (p < 0.001) in the intervention group. The findings show that adding glenohumeral adductor coactivation to a rotator cuff-strengthening program does not result in improved short-term efficacy in any of the outcomes. However, there may be promising results in the medium or long term.
Ingwersen et al.^^14^^	8/10	Patients with rotator cuff tendinopathy were recruited and randomized to 12 weeks of high-load exercise vs low-load exercise and stratified for concomitant administration of corticosteroid injection.	The primary outcome was change from baseline to 12 weeks in the DASH questionnaire, assessed in the intention-to-treat population.	Study results did not show superior benefit from high-load exercise over low-load exercise (p = 0.61) among patients with rotator cuff tendinopathy. Further investigation of the possible interaction between exercise type and corticosteroid injection (p = 0.28) is needed to establish the potential benefits of this combination.
Heron et al.^^15^^	7/10	120 patients with rotator cuff tendinopathy with full range of movement at the shoulder underwent 3 dynamic rotator cuff loading programmes: open chain resisted band exercises, closed chain exercises, and minimally loaded range of movement exercises.	Change in SPADI score.	All three programmes resulted in significant decreases in SPADI score; however, there were no significant differences between the groups (p = 4.0, p = 3.5, and p = 0.5).

DASH = Disabilities of the Arm, Shoulder and Hand; VAS = Visual Analog Scale; PEDro = Phisiotherapy Evidence Database; SPADI = Shoulder Pain and Disability Index; WORC = Western Ontario Rotator Cuff.

Our study results suggest that patients diagnosed with rotator cuff tendinopathy treated with therapeutic exercise have reduced pain and improved shoulder functioning. Furthermore, it was observed that goniometry, visual analogue scales (VAS), the Constant Murley score, the Disabilities of the Arm, Shoulder and Hand questionnaire, and the Shoulder Pain and Disability Index are constantly used to measure pain and functioning in these patients.

According to our results, eccentric exercise, conventional exercise, scapular and rotator cuff muscle strengthening, rotator cuff strengthening plus coactivation with pectoralis major, high-load exercise, and low-load exercise were shown to be effective for the outcomes investigated in this study. However, consistently with Kachingwe et al.,^^2^^ no exercise has been identified as superior in reducing pain and increasing functioning in patients with rotator cuff tendinopathy.

Although all exercise-based treatments investigated in the RCTs appear to be effective in reducing pain and improving functioning in patients with rotator cuff tendinopathy, there were no statistically significant differences between the groups.^^12^-^15^^ Therefore, results from the RCTs included in this review indicate that we currently do not know which exercise-based therapy is most effective in reducing pain and increasing functioning in these patients.

When comparing conventional exercise to eccentric exercise, Dejaco et al.^^12^^ reported pain reduction and improved functioning in both groups after a 12-week daily exercise program. However, there were no statistically significant differences between the groups (p = 0.015).

Boudreau et al.^^13^^ analyzed 42 participants with rotator cuff tendinopathy randomly assigned to scapular and rotator cuff muscle strengthening vs rotator cuff strengthening plus coactivation with pectoralis major and latissimus dorsi recruitment. There were no statistically significant differences between the groups (p = 0.55). However, there were significant group-by-time interactions for the Western Ontario Rotator Cuff questionnaire and VAS (p < 0.001). Therefore, we can assume that rotator cuff strengthening plus coactivation with pectoralis major and latissimus dorsi recruitment may be more beneficial in the long or medium term. Still, this hypothesis needs to be further investigated.

Ingwersen et al.^^14^^ conducted 12 weeks of high-load vs low-load exercises and evaluated baseline changes in the DASH questionnaire. Study results did not show superior benefit from high-load exercise over low-load exercise (p = 0.61).

Heron et al.^^15^^ compared open chain resisted band exercises, closed chain exercises, and minimally loaded range of movement in relation to the Pain Index and the Shoulder Pain and Disability Index and found no statistically significant differences between the groups (p = 4.0, p = 3.5, and p = 0.5).

Our study results suggest that exercise-based interventions in patients with shoulder tendinopathy can reduce pain and improve functioning. However, when choosing the appropriate intervention, the physician must investigate the patient’s clinical criteria, as well as their tolerance and adherence to the exercise.

Although consistent data were found in this study, and given the methodological biases of some studies, we suggest that new RCTs be conducted with the aim of understanding the effect of therapeutic exercise on patients with rotator cuff tendinopathy. Future studies should investigate exercise dosage, kinesiophobia, and pain catastrophizing, as well as develop new assessment tools for this condition. The International Classification of Functioning, Disability and Health should be increasingly used in the clinical evaluation of these patients. In addition, the questionnaires currently used by physicians to assess shoulder disorders should be improved, and their clinimetric properties should be further investigated.

## CONCLUSIONS

Exercise-based therapy (therapeutic exercise) effectively reduces pain and improves functionality in patients with rotator cuff tendinopathy. However, no therapy is currently superior to another. Finally, new RCTs should be conducted to clarify the possibilities of conservative approaches in this population.

## References

[1] Frassanito P, Cavalieri C, Maestri R, Felicetti G. (2018). Effectiveness of extracorporeal shock wave therapy and kinesio
taping in calcific tendinopathy of the shoulder: a randomized controlled
trial. Eur J Phys Rehabil Med.

[2] Kachingwe AF, Phillips B, Sletten E, Plunkett SW. (2008). Comparison of manual therapy techniques with therapeutic exercise
in the treatment of shoulder impingement: a randomized controlled pilot
clinical trial. J Man Manip Ther.

[3] Frantz AC, Stacke BS, Costa J, Gregory J, Brito P. (2012). Efeito do tratamento fisioterapêutico em paciente com
suspeita de síndrome do impacto do ombro: estudo de
caso. Cad Pedagog.

[4] Borges DRSC, Macedo AB. (2010). Os benefícios da associação da laserterapia
e exercícios terapêuticos na síndrome do impacto do
ombro: estudo de caso. Saude CESUC.

[5] Barbosa RI, Goes R, Mazzer N, Fonseca MCR (2008). A influência da mobilização articular nas
tendinopatias dos músculos bíceps braquial e
supra-espinal. Braz J Phys Ther.

[6] Mendonça Jr HP, Assunção AA. (2005). Associação entre distúrbios do ombro e
trabalho: breve revisão da literatura. Rev Bras Epidemiol.

[7] Moher D, Liberati A, Tetzlaff J, Altman DG, PRISMA Group (2009). Preferred reporting items for systematic reviews and
meta-analyses: the PRISMA statement. PLoS Med.

[8] Mancini MC, Cardoso JR, Sampaio RF, Costa LCM, Cabral CMN, Costa LOP. (2014). Tutorial for writing systematic reviews for the Brazilian Journal
of Physical Therapy (BJPT). Braz J Phys Ther.

[9] Lima GCS, Barboza EM, Alfieri FM (2007). Análise da funcionalidade e da dor de indivíduos
portadores de síndrome do impacto, submetidos à
intervenção fisioterapêutica. Psicoter Mov.

[10] Shiwa SR, Costa LOP, Costa LCM, Moseley A, Hespanhol Jr LC, Venâncio R (2011). Reproducibility of the Portuguese version of the PEDro
Scale. Cad Saude Publica.

[11] Ryans I, Galway R, Harte A, Verghis R, Agus A, Heron N (2020). The effectiveness of individual or group physiotherapy in the
management of sub-acromial impingement: a randomised controlled trial and
health economic analysis. Int J Environ Res Public Health.

[12] Dejaco B, Habets B, van Loon C, van Grinsven S, van Cingel R (2017). Eccentric versus conventional exercise therapy in patients with
rotator cuff tendinopathy: a randomized, single blinded, clinical
trial. Knee Surg Sports Traumatol Arthrosc.

[13] Boudreau N, Gaudreault N, Roy JS, Bédard S, Balg F. (2019). The addition of glenohumeral adductor coactivation to a rotator
cuff exercise program for rotator cuff tendinopathy: a single-blind
randomized controlled trial. J Orthop Sports Phys Ther.

[14] Ingwersen KG, Jensen SL, Sørensen L, Jørgensen HR, Christensen R, Søgaard K (2017). Three months of progressive high-load versus traditional low-load
strength training among patients with rotator cuff tendinopathy: primary
results from the double-blind randomized controlled RoCTEx
trial. Orthop J Sports Med.

[15] Heron SR, Woby SR, Thompson DP. (2017). Comparison of three types of exercise in the treatment of rotator
cuff tendinopathy/shoulder impingement syndrome: A randomized controlled
trial. Physiotherapy.

